# Current trends and research topics regarding liver 3D bioprinting: A bibliometric analysis research

**DOI:** 10.3389/fcell.2022.1047524

**Published:** 2022-11-28

**Authors:** Bao Jin, Yitong Liu, Shunda Du, Xinting Sang, Huayu Yang, Yilei Mao

**Affiliations:** ^1^ Department of Liver Surgery, Peking Union Medical College Hospital (PUMCH), Chinese Academy of Medical Sciences (CAMS) & Peking Union Medical College (PUMC), Beijing, China; ^2^ Peking Union Medical College (PUMC), PUMC & Chinese Academy of Medical Sciences, Beijing, China

**Keywords:** 3D bioprinting, liver, current trends, bibliometric analysis, hepatocyte

## Abstract

**Introduction:** Over recent years, 3D bioprinting has changed dramatically. The articles related to liver 3D bioprinting have not been quantitatively analyzed. In this article, we screen all articles related to liver 3D bioprinting until January 2022 and analyzed them using bibliometric citation analysis to characterize the current trends in liver 3D bioprinting.

**Methods:** The articles were identified and analyzed from the Clarivate Analytics Web of Science Core Collection database.

**Results:** Until 1 January 2022, 71 articles focusing on liver 3D bioprinting were identified. There was an increase in the number of articles in 2015. Most articles came from the USA (*n* = 27), followed by South Korea (*n* = 22), China (*n* = 16), and Japan (*n* = 5). The printing technology of liver 3D printing was the most studied topic (*n* = 29). Biofabrication published the highest number of papers (*n* = 16) with 1,524 total citations.

**Conclusion:** Based on bibliometric analysis of the articles until January 2022, a comprehensive analysis of the liver 3D bioprinting articles highlighted the current trends and research topics of this field. The data should provide clinicians and researchers insight into future directions relative to the liver 3D bioprinting.

## 1 Introduction

The liver is a major metabolic organ in the body and plays an important physiological role in bile secretion, blood clotting, and immunity. Meanwhile liver is also an important organ for detoxification of exogenous or endogenous substances in the body. Although the liver has a strong regenerative capacity, when liver cell damage exceeds a certain level, the regenerative capacity of liver cells is disrupted, resulting in irreversible damage to liver cells. Severe impairment of liver function is mainly caused by various causes of liver diseases as well as drug-induced liver injury. Therefore, *in vitro* hepatocyte models are an important way to study the physiological functions of hepatocytes and to perform drug screening (Guguen-Guillouzo et al., 2010; Bhatia et al., 2014). In addition, liver transplantation is an effective treatment for various end-stage liver diseases. However, the problem of organ shortage is becoming more and more prominent, and many researchers have proposed the use of tissue engineering techniques for *in vitro* culture of tissues and organs to solve the problem of solid organ donor shortage. Therefore, *in vitro* hepatocyte models are an important basis for conducting liver cultures.

Before the advent of 3D printing technology, *in vitro* cell experiments were often performed using 2D culture systems and common 3D culture systems. Among them, the traditional 2D cell line model is overly simplistic and severely detach from the biological system of the organism in culture, ignoring the fact that *in vivo* hepatocytes are 3D structures both morphologically and histologically. Sandwich culture was an early 3D culture method used (Swift et al., 2010). The morphology of hepatocytes in this culture system is flatter compared to hepatocytes *in vivo*. Meanwhile the cells lack cell-to-cell interactions, while the maintenance of albumin and related enzyme secretion in hepatocytes is relatively short (Berthiaume et al., 1996). Therefore, the results of physiological and pharmacological studies of hepatocytes using this culture mode are not satisfactory. In recent years, 3D bioprinting technology has developed rapidly. 3D printing is also known as Additive Manufacturing (AM). It is based on computer modeling and combines material processing and molding technologies to build three-dimensional structures by stacking materials layer by layer through different methods. Among them, 3D bioprinting is the most cutting-edge field of 3D printing technology, using cells and biological materials as bio-ink, and precisely controlling the spatial layout of cells and surrounding microenvironment according to pre-design, which simulates the real environment *in vivo* to the maximum extent and provides new possibilities for building *in vitro* hepatocyte experiments (Sun et al., 2020a). The 3D bioprinting studied in this paper refers to 3D printing using living cells.

Bibliometric citation analysis is a widely used method of literature mapping analysis that can help us understand where 3D bioprinting is headed in the future. The citation number of an article indicates the importance of the study and reflects the direct impact on the understanding and treatment of the disease. In this study, we analyzed all past articles on liver 3D printing to provide an overview of the development of 3D bioprinting. In turn, these data provide clinicians and researchers with a comprehensive understanding of evolution and meaningful insights into the future direction of 3D bioprinting.

## 2 Methodology

### 2.1 Literature search and screening

Until January 2022, The Clarivate Analytics Web of Science Core Collection database was systematically searched. A summary of key words and the search strategy is shown in online Supplementary Table S1. Only original articles which focused on the 3D bioprinting of liver were included. Reviews were excluded; meeting abstracts, editorial materials, book chapters, and were also excluded to limit selection of articles to only those with high scientific merit in the field. Two reviewers (B.J. and Y.T.L.) independently identified the articles, and any disagreement between the 2 reviewers was resolved by consensus involving a third reviewer H.Y.Y.

### 2.2 Data analyses and visualization

After identifying all the articles, we downloaded the records and cited references including all available information from the Web of Science Core Collection database. The bibliographic information of the selected publications was converted and analyzed automatically by using the bibliometric package (Version 3.0.0) in R software (Version 3.6.1), as reported previously (Aria and Cuccurullo, 2017). The information was extracted and analyzed using the bibliometric package including authors, title, countries, institutions or regions, year of publication, number of TC, impact factor and journal. The main topic, sub topic, and article type of each article were also determined by reading the title, abstract, and full text, if necessary.

All the information and data for each article were inserted into a spreadsheet and manipulated using Microsoft Excel 2019 (Microsoft Corp., Redmond, WA, USA). We created the graphs and figures using R software (Version 3.6.1) and EChart.js package (Version 4.5.0; https://echarts.apache.org/en/index.html), which is based on JavaScript.

## 3 Results

### 3.1 Publication period and citation count

**FIGURE 1:**
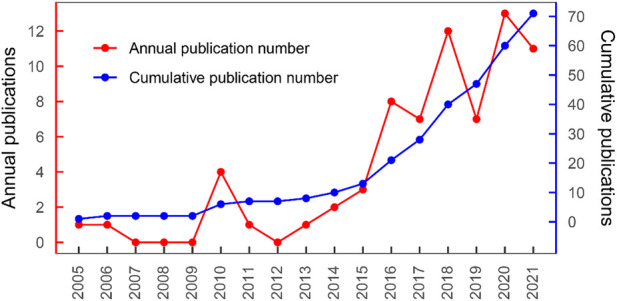
Literature growth curve.

Until 1 January 2022, 71 articles focusing on liver 3D bioprinting were identified by consensus in the Web of Science Core Collection database. All articles are listed in online Supplementary Table S2 in descending order according to the article’s TC number. The number of citations varied from each paper and ranged from 565 (“The 3D printing of gelatin methacrylamide cell-laden tissue-engineered constructs with high cell viability”) to 0 (“Bile duct reconstruction using scaffold-free tubular constructs created by Bio-3D printer”). Figure 1 presents the annual number of publications and cumulative number of publications. Between 2005 and 2015, the literature grew slowly, whereas the number of published articles increased relatively fast over the past 6 years. The earliest article in the list, which focused on printing technology of liver 3D bioprinting, was published on BIOMATERIALS in 2005 (Yan et al., 2005). The first author of this article was Yongnian Yan from Tsinghua University. The researchers developed an organ manufacturing technique that enables to form cell/biomaterial complex three-dimensional (3D) architectures in designed patterns. As of February 2022, this article has been cited 211 times in the WOS Core Collection database.

### 3.2 Countries or regions, institutions, and authors

**FIGURE 2:**
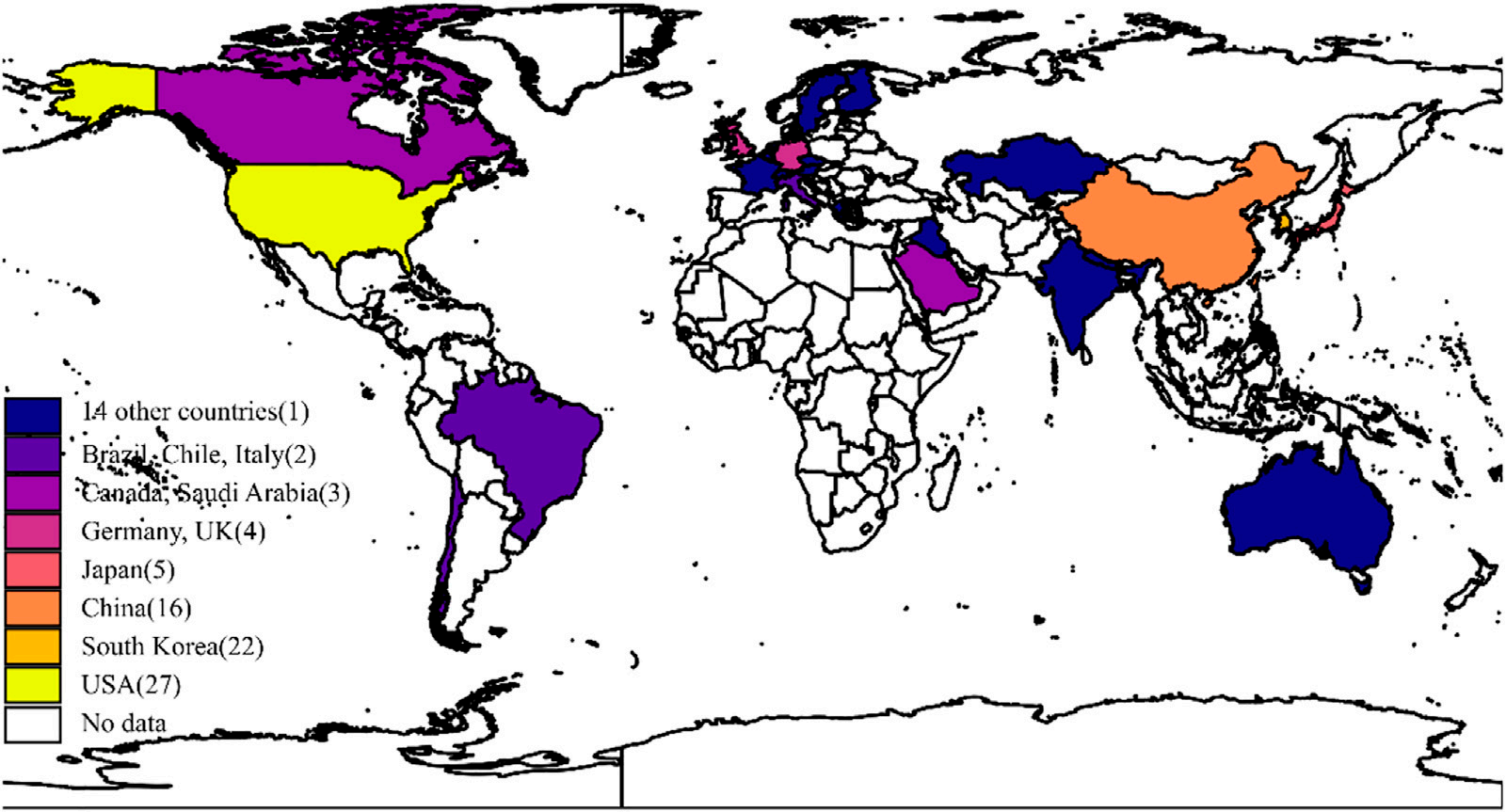
Number of publications by country or region.

**FIGURE 3:**
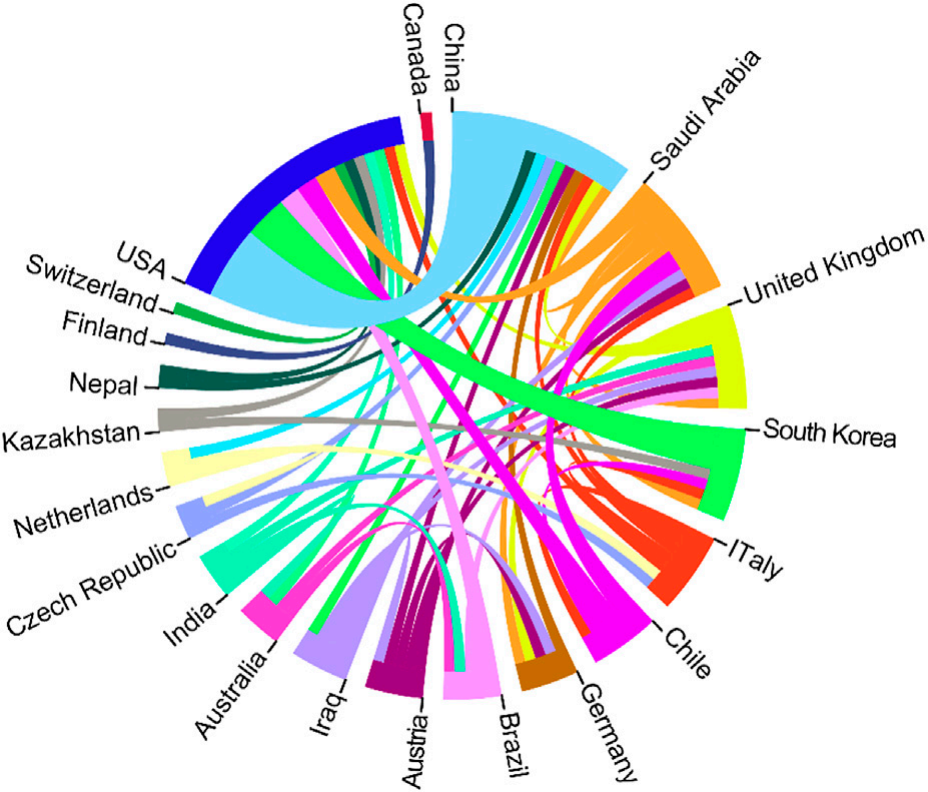
The cooperation relationship of countries or regions that published articles.

In analyzing countries (or regions) and institutions of the authors, the articles were originated from 25 countries or regions (shown in Figure 2). Three countries and regions contributed >10 articles, and 14 countries contributed 1 article (shown in online Supplementary Table S3). Among all the articles, the USA contributed the most articles (*n* = 27), follow by South Korea (*n* = 22), China (*n* = 16), Japan (*n* = 5), Germany (*n* = 4), and UK (*n* = 4). Figure 3 depicts the partnership among countries that published articles, which demonstrated close cooperation among the various countries and regions.

**FIGURE 4:**
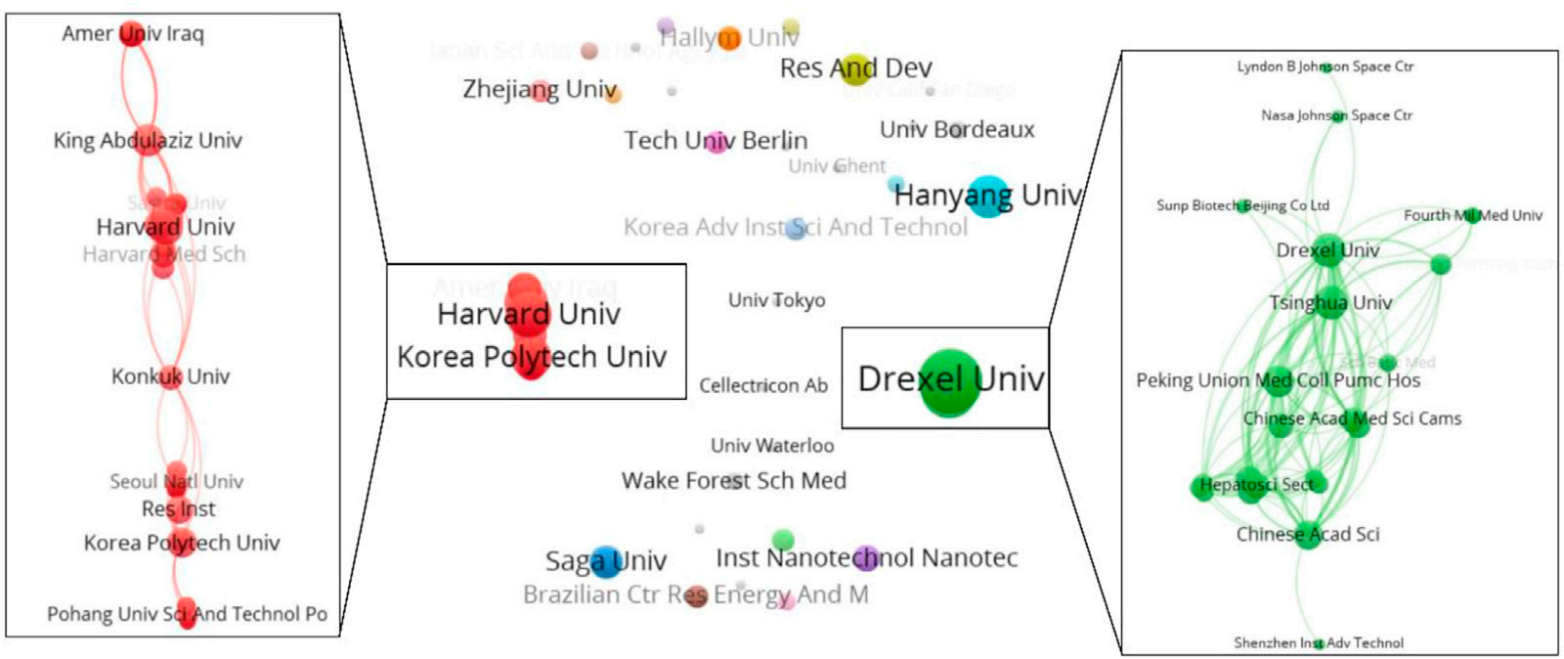
The cooperation relationship of institutions that published articles.

**FIGURE 5:**
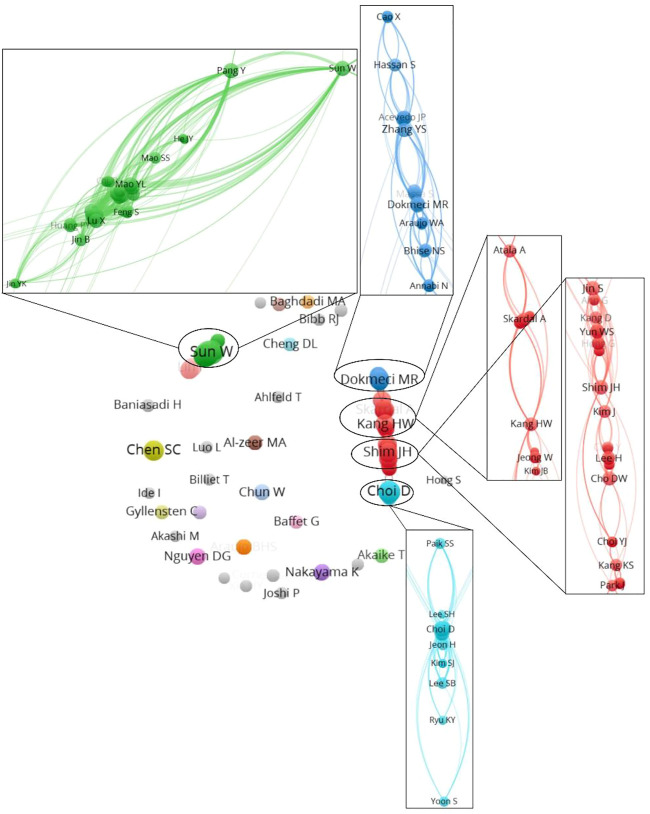
The cooperation relationships of authors that published articles.

As noted in Table 1, the top 10 institutions which published the most articles included the Tsinghua University, Drexel University, and Korea Polytechnic University with 8, 7 and 5 papers, respectively, with 634 citations, 348 citations, and 192 citations, respectively. The ratio of TC to publications reflects the TC numbers of each article. Harvard University has the highest ratio of TC (796), followed by the Tsinghua University (634) and University of California San Diego (571) (shown in Table 1). The cooperation among different countries, institutions, and authors is a critical driving force to promote the development of most successful large-scale trials. To this point, there seemed to be close cooperation among different institutions from various countries and regions (Figures 3, 4). Moreover, authors were classified into >10 clusters in the authors’ collaboration network analysis; several major research teams were identified, mainly including Sun W, Dokmeci MR, Atala A, Kang HW, Shim JH, Kang KS, Choi D, Chen SC (shown in Figure 5).

**TABLE 1:** Top 10 institution with the most publications.

Institutions	Publication	TC	TC/Publication
Tsinghua University	8	634	79.25
Drexel University	7	348	49.71
Korea Polytechnic University	5	192	38.40
Pohang University of Science and Technology	5	453	90.60
Chinese Academy of Sciences	4	60	15.00
Hanyang University	4	117	29.25
Harvard University	4	796	199.00
Korea Institute of Machinery and Materials	4	117	29.25
Peking Union Medical College Hospital	4	63	15.75
University of California San Diego	4	571	142.75

TC, total citation.

### 3.3 Journals

The articles were published in 40 journals. According to the number of the articles published, the top 10 journals are listed in Table 2. Biofabrication published the articles with 16 papers. Biofabrication generated the largest quantity of TC with 1,524 citations. There were 3 journals with the ratio of TC to publications exceeding 100, namely, Biomaterials (TC/publications 174.43), Tissue Engineering Part A (TC/publications 148.00), and Plos One (TC/publications 109.50).

**TABLE 2:** Top 10 journals with the most publications.

Journal	Publication	IF	TC	TC/publication
Biofabrication	16	10.020	1,524	95.25
Biomaterials	7	12.479	1,221	174.43
Scientific reports	5	4.380	68	13.60
Advanced healthcare materials	3	9.933	201	67.00
Advanced Materials	2	30.849	123	61.50
Genes	2	4.096	77	38.50
Plos one	2	3.240	219	109.50
Tissue engineering part A	2	3.845	296	148.00
ACS applied materials & interfaces	1	9.229	31	31.00
ACS biomaterials science & engineering	1	4.749	7	7.00

IF, impact factor; TC, total citation.

### 3.4 Topics

Of the 71 articles so far, they can be broadly classified into 3 categories based on the topic, which are exploring printing technology, studying bio-ink, and biological applications. As showed in Table 3 (Kim et al., 2020), articles examine printing technology, 19 articles examine bio-ink, and 23 articles examine biological applications. The first articles on liver 3D bioprinting was published in 2005 by XiaoHong Wang’s team at Tsinghua University in BIOMATERIALS (Yan et al., 2005). This is the first step in the development of an implantable bioartificial liver. Among the nine recently published articles, studies on printing technology and biological applications accounted for eight of them.

**TABLE 3:** The number of different topics.

Topic	Number of documents	Ratio (%)
Printing technology	29	40.8
Bio-ink	19	26.8
Biological application	23	32.4

**FIGURE 6:**
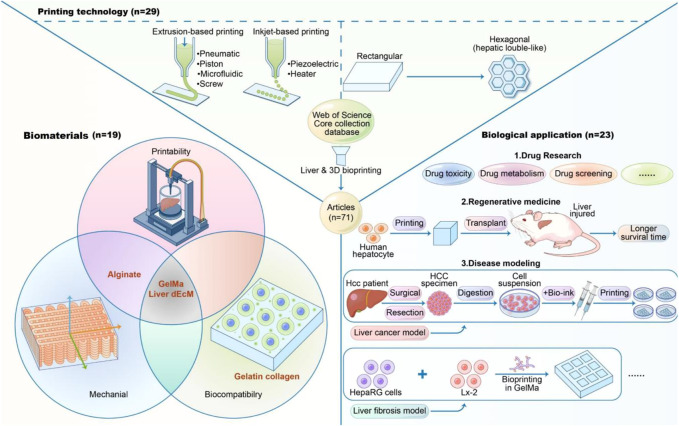
Abstract figure.

As shown in Figure 6, the discussion of topic in this review is roughly sketched.

When categorizing articles thematically, the authors classified them according to which aspect the article focused more.

#### 3.4.1 Printing technology

3D bioprinting technology has developed relatively quickly and a variety of printing methods have emerged, the most used in liver-related 3D printing articles are extrusion-based printing and inkjet printing. Among them, inkjet 3D bioprinting is the earliest bioprinting technology used in the screen-out articles, and the basic principle is similar to 2D printing, which a droplet of bio-ink is first printed to a set specific location, followed by cross-linking and gelation between the droplet and the substrate upon contact (Angelopoulos et al., 2020). This printing method has a high resolution, which can achieve single cell level, and is extremely cost effective, but this approach is limited by the choice of bio-ink and the number of cells, and the print nozzle is usually limited to a low viscosity material to prevent clogging of the nozzle. In addition the relatively slow printing speed due to the point-by-point deposition approach has resulted in fewer applications in building more complex tissue models. Inkjet bioprinting is a method for constructing liver tissue scaffolds. The use of biocompatible surfactants can enhance the ability of HepG2 cells to form droplets for inkjet bioprinting (Parsa et al., 2010). Arai et al. (2017) constructed a 3D culture system of hepatocytes using alginate hydrogel as a scaffold by droplet-based printing technique, preserving the specific functions of hepatocytes.

Extruded 3D printing is currently the most used approach when constructing liver tissue models. This is done by encapsulating cells in a bio-ink and then extruding the biomaterial into a continuous filamentary form by external extrusion and stacking it layer by layer into a three-dimensional structure. Extruded 3D bioprinting bio-ink are more compatible and can be selected from a wide range of options, including suspensions, decellularized extracellular matrix solutions and hydrogels with a wider range of viscosities, such as polyethylene glycol-based hydrogels, gelatin, hyaluronic acid and alginates (Jia et al., 2016; Ozbolat and Hospodiuk, 2016; Rastogi and Kandasubramanian, 2019). The multiple selective bio-ink make extruded 3D bioprinting more flexible compared to inkjet printing, allowing the construction of a variety of heterogeneous tissue models.

Wu et al. used micro-extrusion-based 3D printing technology to construct hepatic lobule-like mock structures using a combination of hepatocellular carcinoma cell lines and fibroblast cell lines (NIH/3T3) and two types of bio-ink, and showed that complex constructs with multiple cell types and different extracellular matrices could be constructed by an extrusion-based 3D printing process, and that the multicellular liver constructs exhibited albumin enhanced secretion (Wu et al., 2020). It has been shown that extrusion-based 3D printing can construct vascularized liver lobule structures by pre-designing and printing liver lobule-shaped pre-designed cartridges containing three chambers, namely hepatocyte chambers, endothelial cell chambers, and hollow chambers. And multicellular vascularized liver lobule structures were constructed by this pre-designed extrusion-based bioprinting, and the engineered liver lobules have synthetic, secretory, and drug metabolism (Kang et al., 2020). Although extrusion printing also has advantages over inkjet printing in terms of cell viability, bio-ink deposition speed, and scale up. However, the resolution of extrusion printing can be relatively low and needs to be improved (Ma et al., 2020).

Meanwhile, bioprinting in a core-shell fashion is also a promising option to spatially determine the arrangement of several cell types. Two or even more bio-inks can be simultaneously extruded through a coaxial needle to form strands with two discrete compartments, with an internal core completely enclosed within an external (possibly stable) shell (Perez and Kim, 2015). Thus, this technique could in principle imprint different cell types together so that they interact with each other. Taymour et al. (2021) have established a functional co-culture model with independently adjustable compartments for different cell types by core-shell bioprinting. This provides the basis for more complex *in vitro* models that allow hepatocytes to be co-cultured with other liver-specific cell types to closely resemble the liver microenvironment.

In addition, the print shape set during the printing process is mainly rectangular, while some articles print in a circular or hepatic lobule-like hexagonal shape. The basic building block of the liver is the hepatic lobule, which resembles a square hexagon with a central vein in the center and a portal vein containing the confluent area at the edge, and the hepatocytes are polarized (van Grunsven, 2017; Ma et al., 2020). *In vitro* fabrication of liver lobules is challenging, and 3D bioprinted liver tissue is gradually progressing from shapeless to hexagonal shapes that mimic liver lobules. In the rectangular model, when the 3D bioprinted liver tissue is large, the central region may be in a hypoxic and undernourished state, resulting in slow and weak metabolism of drugs and toxins (Kang et al., 2021). So kang et al. constructed a hexagonal bioprinted liver construct and combined rotational regulation with continuous media stimulation and found that HepG2 exhibited enhanced proliferative capacity and biological function. It has also been shown that the geometry of the liver construct may affect liver function. Lewis et al. found that the angle of the extruded 3D bioprinted grid lines affected liver tissue function, as evidenced by the fact that liver tissue with a 60° angle secreted better levels of albumin than liver tissue with a 90° angle, as well as higher gene expression and activity of CYP3A4 and CYP2C9 than the latter, suggesting that 3D bioprinting pattern conformation on liver tissues not only in appearance mimicry, but also affects functional mimicry (Lewis et al., 2018).

#### 3.4.2 Bio-ink

Bio-ink contains two parts, which are biomaterials and cells. During actual printing process, these two parts are mixed and printed according to different proportions and densities, and then fixed under different curing conditions or not to obtain the desired 3D-printed bodies for further related experiments.

##### 3.4.2.1 Biomaterials

Biomaterials are used to encapsulate cells in 3D bioprinting and need to have good printability, biocompatibility and mechanical properties. Printability is the assessment of the formability of the bio-ink, which needs to have adjustable viscosity, fast transition from solution to gel state and a wide range of printing parameters (Gu et al., 2020). Biocompatibility requires that the bio-ink resembles the real microenvironment in the body as much as possible, and that the bio-printed small can maintain high viability and biological function without affecting the diffusion of nutrients and oxygen. Mechanical properties are required for the bio-ink to maintain a good 3D structure after 3D printing for subsequent *in vitro* culture and *in vivo* inhibition. Therefore, balancing printability, biocompatibility and mechanical properties is a key consideration for bio-ink development.

In the screened liver-related 3D printing articles, the commonly used bioinks are alginate, GelMA, Gelatin, collagen, etc. The commonly used curing methods are CaCl2, LAP + UV, temperature, etc., which are cured in different ways at different stages of printing to form gel-like constructs (Heinrich et al., 2019). Among them, Alginate is the most used bio-ink with good printability and mechanical properties, generally fixed by CaCl2, with the relative shortcoming of weak biocompatibility, which affects cell adhesion. Gelatin is a water-soluble protein extracted from connective tissue, which is the main component of human tissue and contains a variety of amino acids required by the human body, thus its biocompatibility is better. GelMA has become a popular bio-ink in recent years because of good printability, biocompatibility and mechanical properties. Bertassoni et al. (2014) bioprinted cell-loaded GelMA at 7%–15% and found that the encapsulated HepG2 cells maintained cell viability for at least 8 days after the bioprinting process. Grix et al. (2018) used GelMA as bio-ink loaded with HepaRG cells and human hepatic stellate cells to construct liver-like organs by DLP-based bioprinting and found that 3D printed liver tissue had better liver function compared to 2D cultured cells. Collagen is a water-insoluble fibrous protein in the extracellular matrix with good biocompatibility, but its mechanical properties are poor, and collagen scaffolds are prone to collapse, and their mechanical properties can be enhanced by combining with other biomaterials. Mazzocchi et al. (2018) investigated the properties of bio-ink containing different ratios of collagen and hyaluronic acid, and found that liver tissue constructs could maintain albumin secretion and urea synthesis, and that the drug response of liver constructs to acetaminophen (APAP) could be maintained for 2 weeks when the collagen/hyaluronic acid ratio in the scaffold was 3/1. In addition, a recent study has developed liver dECM as a specific bio-ink for 3D printing of liver tissue. Compared with other bio-ink, liver dECM bio-ink not only retains the major components of liver extracellular matrix and enhances its biocompatibility, but in addition, its printability and mechanical properties are significantly improved (Lee et al., 2017; Ma et al., 2018; Lee et al., 2019; Yu et al., 2019; Kim et al., 2020; Lee et al., 2020; Mao et al., 2020; Jeong et al., 2021).

##### 3.4.2.2 Cells

**FIGURE 7:**
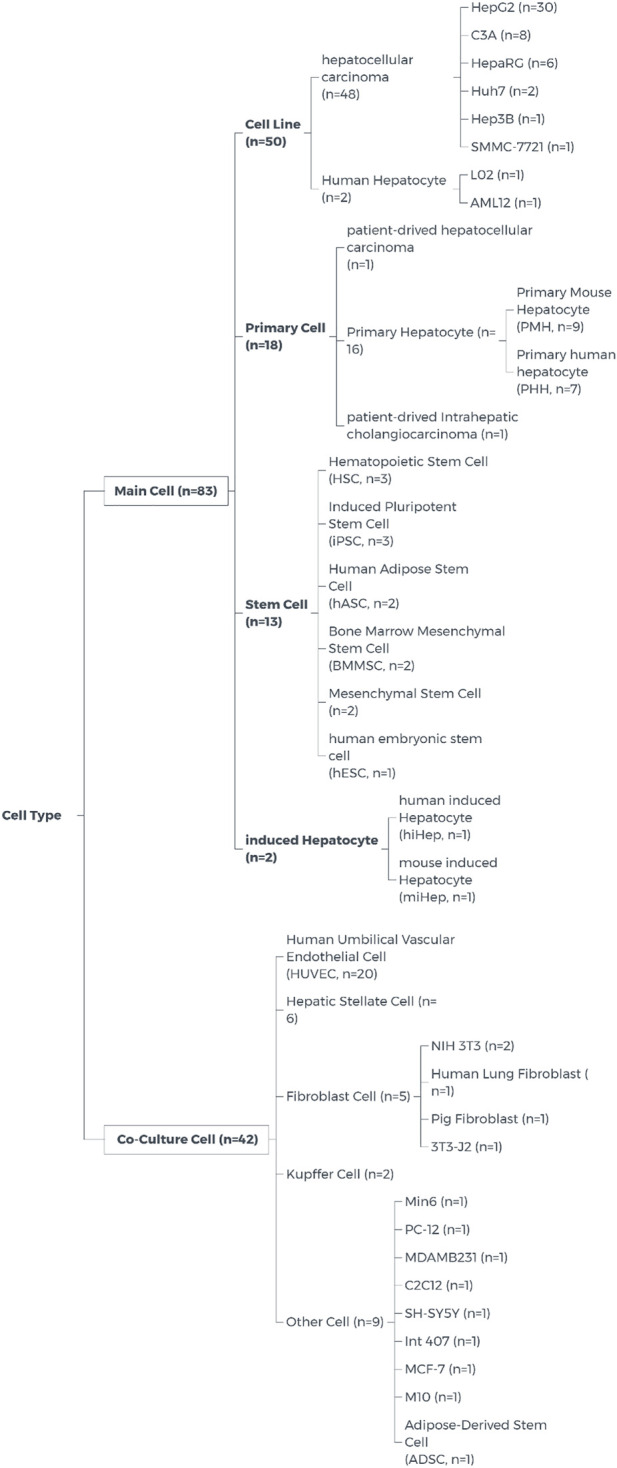
Cell type on liver 3D Bioprinting.

In the selected articles on 3D bioprinting, the selection of cells can be divided into single cell printing and multicellular printing according to the number of cells types. For single cell printing, HepG2, HepaRG, PMH and so on are the main cells used. Some studies use these single cells to test the biocompatibility of bioink (Baniasadi et al., 2021), and some research on printing methods (Hong and Song, 2021). In multicellular printing, two types of cells are mainly used, main cells and co-culture cells. Main cells refer to cells that have the role of hepatocytes or can be differentiated into hepatocytes. There are four main categories: cell line, primary cell, stem cell, induced hepatocyte. Co-culture cells refer to cells that are mixed with or printed near main cells, mainly to better mimic the liver environment *in vivo*. They mainly include human umbilical vascular endothelial cells (HUVEC), fibroblast cells, and fibroblast cells. Kupffer cell and so on. The specific cell classification of each item can be seen in Figure 7.

When talking about the choice of cells, many researchers tend to use cell lines, because they are usually high proliferation and easier to cultivate and transfection. Most of the cell line has been training for decades, which can better adapt to the conditions. However this led to cell lines are genetically and phenotypically different from tissue origin (American Type Culture Collection Standards Development Organization Workgroup ASN, 2010; Lorsch et al., 2014). Primary cells often have normal cell morphology and maintain many important markers and functions *in vivo*, which can better simulate the environment *in vivo*. However, comparing with cell lines, primary cells isolated directly from tissues often have a limited lifespan, limited expansion capacity, and require additional nutrients which are not available in conventional culture media. This has hindered researchers from using primary cells for research. Although it may be more difficult to use primary cells, the data obtained using primary cells are more relevant and reflective of the *in vivo* environment (Alge et al., 2006; Pan et al., 2009). Optimizing the culture conditions of primary cells has become an urgent problem to be solved for the use of primary cells for 3D bioprinting.

#### 3.4.3 Biological application

Among the screened liver-related 3D bioprinting articles, the biological application aspects can be mainly divided into drug research, regenerative medicine, and disease modeling.

Among the drug studies include studies on drug toxicity, drug metabolism, drug screening, etc. Gori et al. (2020) applied 3D bioprinted liver tissue models for drug toxicity studies. They constructed 3D bioprinted liver tissue from HepG2/C3A using Pluronic/alginate semisynthetic hydrogels and demonstrated the high viability and liver-specific metabolic activity of the printed bodies. It was also found that the sensitivity of 3D printed bodies to the hepatotoxic drug acetaminophen was significantly increased compared to 2D cultured cells, making 3D liver tissue models an alternative to *in vitro* animal models for studying drug-induced hepatotoxicity. Similarly, Lee et al. and Bhise et al. developed liver constructs by 3D bioprinting which exhibited similar drug response to acetaminophen (Bhise et al., 2016; Lee et al., 2019).

In regenerative medicine, Zhong et al. (2016) constructed a hydrogel scaffold containing the human normal hepatocyte line HL-7702 and transplanted it into liver-injured nude mice, and found that the liver function of the transplanted group of nude mice was superior to that of the control group, and the survival time was prolonged compared to the control group, which revealed the great potential of 3D bioprinting for liver function reconstruction and liver tissue regeneration. Kang et al. (2018) constructed 3D liver tissue model using mouse embryonic fibroblast-transformed hepatocytes (miHep), and the expression of liver-specific markers albumin, ASGR1, and HNF4a gradually increased during *in vitro* culture. And increased proliferation and higher albumin expression were observed when this construct was transplanted into liver-injured mice. This work demonstrates the potential of using 3D bioprinted liver stent as an effective option for liver regeneration therapy. Yang et al. (2021) constructed a liver tissue model using HepaRG cells, which were demonstrated to have albumin secretion, drug metabolism, and glycogen storage under *in vitro* culture conditions, and subsequently transplanted into Fah gene-deficient chronic liver-injured mice, which acquired human-specific drug metabolism activity, and transplanted prints in which also formed functional vascular system that enhanced substance transport and liver function. Most importantly, the results showed transplant significantly improved the survival of mice with chronic liver failure.

In addition, 3D bioprinting technology has promoted the improvement and application of hepatocellular carcinoma models. Many researchers have constructed single-cell liver cancer models such as hepatocellular carcinoma cell lines HepG2 and Huh7 based on extrusion-based 3D bioprinting. The hepatocellular carcinoma cell lines maintain high viability and retain some functions associated with hepatocytes and were used for liver tissue studies in early studies (Billiet et al., 2014; Jeon et al., 2017; Lewis et al., 2018). Further, Sun et al. characterized the differences between 3D bioprinted hepatocellular carcinoma models and traditional 2D culture models in detail and found that 3D bioprinted hepatocellular carcinoma models have unique gene expression profiles, not only superior to 2D cells in the expression of liver function-related genes, but also enhanced expression of tumor-related characteristic genes, such as proliferation, invasion, stemness, and autophagy (Sun et al., 2020b). In addition, Xie et al. (2021) also made a breakthrough in constructing individualized liver cancer models using 3D bioprinting. They constructed six individualized tumor models of liver cancer patients using 3D bioprinting technology and found that the 3D bioprinted primary liver cancer tissues retained the characteristics of the primary generation such as gene mutation profile, biomarker expression and specific gene expression profile, demonstrating the value of 3D bioprinting in clinical precision therapy applications.

In parallel, advances in 3D bioprinting of liver tissue have led to the development of liver fibrosis models. Norona et al. (2019) used 3D bioprinting of liver tissue containing primary hepatocytes, hepatic stellate cells, and endothelial cells in a typical activated state of stellate cells to mimic compound- and factor-induced liver injury and fibrosis by repeated low-level exposure to methotrexate, thioacetamide, and TGF-β1. Cuvellier et al. (2021) constructed lattice-like liver fibrosis models using hepatic parenchymal cells HepaRG, LX-2, and HUVEC, which were able to settle on the surface of the structures and reconstruct endothelial-like barriers, while protofibrillar collagen deposition was observed. Thus 3D bioprinted liver fibrosis models provide a new platform to study the mechanisms of fibrosis development.

## 4 Discussion

In recent years, 3D bioprinting technology has developed rapidly, going through different stages from the printing of simple biological materials to the printing of living cells, from the printing of pure biomaterials to the printing of living cells. 3D printing of living cells uses cells and biomaterials as bio-ink, which can precisely control the spatial layout of cells and surrounding microenvironment to maximize the simulation of the real environment *in vivo*, and is used to build *in vitro* tissue or organ models, which has great potential for application in organ reconstruction, drug screening, mechanism research, etc. (Sun et al., 2020a).

As mentioned earlier, researchers have been actively exploring the use of 3D bioprinting technology for more than a decade, resulting in breakthroughs in 3D bioprinted liver tissue from scratch, from simple to complex, but there is still much more for exploration. Among them, most of the initial 3D bioprinting used only single cells as cell seeds to verify the feasibility of 3D bioprinted liver tissues, for example, several groups constructed HepG2 lattice-like liver tissues using extrusion-based 3D bioprinting. Jeon et al. (2017) found that HepG2 could proliferate stably in the model constructed by this method, and the expression of genes such as albumin, CYP1A2 and tyrosine aminotransferase in liver tissues was enhanced gradually with the culture time for 14 consecutive days. Zhong et al. (2016) Used the human hepatocyte line LO2 to print lattice-like liver tissue, which showed no significant difference in proliferation from 2D at 1, 3, 5, and 7 days *in vitro*. Cai et al. constructed *in vitro* hepatocytes using light-cured 3D bioprinting and human hiHep cells (derived from human fibroblast transdifferentiation), and survival staining showed that most of the cells were in good condition (Mao et al., 2020). Using HepRG cells, Yang et al. (2021) printed lattice-like models as cell seeds, and after a modified process, cell viability was maintained at about 90% from days 1–10 and up to more than 80% after 3 weeks. These single-cell 3D bioprinting have investigated the feasibility of 3D bioprinting and have made an active exploration for the continuous improvement of the process. There is a trend from single cell printing to multicellular printing, and more research is currently being conducted on the construction of multicellular liver tissue. In co-cultures of two types of cells, the most added non-parenchymal cells are vascular endothelial cells, the addition of which contributes to tissue vascularization (Rouwkema and Khademhosseini, 2016). In addition, umbilical vein endothelial cells are the most widely used, followed by stellate cells, which play a key role in liver fibrosis (Grix et al., 2018). Lewis et al. (2019) co-printed mouse bile duct epithelial cells and human Huh7 cells to form liver tissue with bile ducts. Nguyen et al. used human primary hepatocytes/HepaRG, stellate cells and umbilical vein endothelial cells to co-create liver tissue (Nguyen et al., 2016; Cuvellier et al., 2021). In addition, as in the previous discussion about the choice of liver related aspects, although the primary cell life and expansion capacity are limited, and require additional nutrients which are not available in conventional culture media, the primary cell can better simulate the environment of the organism (Alge et al., 2006; Pan et al., 2009). Therefore, it is necessary to solve these shortcomings and use primary cells for 3D bioprinting research.

Before the advent of 3D bioprinting, 2D culture systems and 3D culture systems were often used for constructing *in vitro* tissue or organ models, conducting drug screening, and studying physiological mechanisms. Compared with the latter, 3D bioprinting has obvious advantages. Among them, the 2D culture system is simple to operate and the technology is more mature, so it is widely used in early research. An important principle of *in vitro* cell culture is the need to mimic the *in vivo* cell growth environment as much as possible, but traditional 2D cell line models are overly simplistic and severely detached from the biological system of the organism in culture, ignoring the fact that in real *in vivo* hepatocytes are 3D structures both morphologically and histologically. It has been shown that the biological behavior and cell-cell information exchange of hepatocytes are greatly affected in a 2D culture system (Treyer and Musch, 2013). In contrast to 3D bioprinting, traditional 2D cell culture lacks complex cell-cell interactions and cell-matrix interactions, and cells grow as a monolayer on the culture dish. Also, it has been found that cells cultured in 3D structures express more liver-specific genes and have better liver architecture than cells cultured in 2D structures (Sun et al., 2020b).

In addition, sandwich culture was an early 3D culture method used. The sandwich culture method was the first 3D culture method developed, which placed tumor cells in a 3D environment and overcame the growth space limitations of 2D flat culture (Swift et al., 2010). However, this culture method still has limitations. Despite more available space, the morphology of hepatocytes in this culture system is flatter compared to hepatocytes *in vivo*, and the cells lack gap junctions and do not establish spatial structure between each other, lacking cell-to-cell interactions, as well as the relatively short maintenance of albumin and related enzyme secretion by hepatocytes (Berthiaume et al., 1996). Therefore, the physiological and pharmacological studies of hepatocytes using this culture model are not satisfactory.

Meanwhile, organoid has become a hot topic of research, and researchers have constructed various bionic tissue and organ models by inducing stem cells to grow into organoids *in vitro*, which has led to great progress in tissue engineering, and tumor organoids derived from primary tumor stem cells have become a hot topic in oncology research (Drost and Clevers, 2018). Since organoids must be cultured from stem cells through a complex induction process, it is relatively complicated to conduct relevant studies using this system. In addition, the culture system requires various expensive growth factors and small molecule compounds, resulting in a high cost of the culture process, and more importantly, the diameter of the induced organoid varies due to the *in vitro* suspension culture, so the pharmacodynamic results obtained in drug screening may be unreliable and poorly reproducible.

There have been many studies using 3D bioprinting to construct liver tissues. For example, a related study used a 3D bioprinting system to construct liver structures from HepG2 cells, and after 3 weeks of culture, the expression of liver-specific markers was quantified by histological, immunohistochemical assays at days 1, 7, 14, and 21, and it was found that compared to 2D culture, the cells in 3D culture grew well (Jeon et al., 2017). In addition, Yang et al. (2021) used HepaRG cells and bioink to construct liver tissues according to a specific 3D printing procedure. And transplanted them into Fah-deficient mice with chronic liver failure and found that 3D-printed bodies acquired a wide range of liver functions such as albumin secretion, drug metabolism and glycogen storage after 7 days of differentiation. The survival rate of Fah-deficient mice transplanted with 3D-printed bodies was also significantly improved, demonstrating that the 3D bioprinted constructed liver tissue has *in vivo* liver function.

In contrast, 3D bioprinting technology has greater advantages when cells are grown under 3D conditions, both in terms of cellular arrangement and expression of cell-associated genes, more closely resembling biological behavior *in vivo*. Many 3D printed culture systems are now thought to predict cellular responses more accurately than 2D culture. 3D bioprinting technology is used in the liver field primarily for regenerative medicine, disease modeling and drug research.

As mentioned above, there is a growing organ shortage and tissue-engineered livers have the potential to be a promising alternative. In addition, personalized drug screening for specific patients still needs to be addressed. Currently, 2D cell culture and animal models are commonly used for new drug development and testing, but 2D cell culture cannot simulate the 3D environment *in vivo*, and animal models are expensive, time-consuming, and differ from human metabolism (Arrowsmith and Miller, 2013). Therefore, it is necessary to develop a new model to simulate the real environment of human liver to achieve stable drug development and testing, and to facilitate research related to liver diseases. 3D bioprinting, an emerging technology, may be a possible alternative to organoid and other bioartificial liver systems, characterized by a high degree of refinement and feasibility in reconstructing the desired tissue or organ, which could potentially replace missing donors in the future or serve as a bridge in the liver transplantation process (Xie et al., 2020).

## 5 Conclusion

Since the first article on 3D bioprinting of the liver appeared in 2005, the authors have sifted through only 71 articles on 3D bioprinting of the liver (Yan et al., 2005). And as mentioned earlier, research on 3D bioprinting in the liver field is still in the preliminary exploration stage. Of these 71 articles, roughly two-thirds are explorations of printing technology, bio-ink, and very few biological application. At present, we can initially apply 3D bioprinting technology to liver-related printing, and preliminarily conduct printing block construction, drug screening, and mechanism research. However, limitations related to the printing process, bio-ink, and cell source still exist during the experimental process. Although the application of liver bioprinting in transplantable functional liver (artificial) organs is still a long way off and needs further development and expansion, the current research shows that liver 3D bioprinting is promising.

## Data Availability

The original contributions presented in the study are included in the article/Supplementary Material, further inquiries can be directed to the corresponding authors.

## References

[B1] Alge C. S. Hauck S. M. Priglinger S. G. Kampik A. Ueffing M. (2006). Differential protein profiling of primary versus immortalized human RPE cells identifies expression patterns associated with cytoskeletal remodeling and cell survival. J. Proteome Res. 5 (4), 862–878. 10.1021/pr050420t 16602694

[B2] American Type Culture Collection Standards Development Organization Workgroup ASN (2010). Cell line misidentification: The beginning of the end. Nat. Rev. Cancer 10 (6), 441–448. 10.1038/nrc2852 20448633

[B3] Angelopoulos I. Allenby M. C. Lim M. Zamorano M. (2020). Engineering inkjet bioprinting processes toward translational therapies. Biotechnol. Bioeng. 117 (1), 272–284. 10.1002/bit.27176 31544957

[B4] Arai K. Yoshida T. Okabe M. Goto M. Mir T. A. Soko C. (2017). Fabrication of 3D-culture platform with sandwich architecture for preserving liver-specific functions of hepatocytes using 3D bioprinter. J. Biomed. Mat. Res. A 105 (6), 1583–1592. 10.1002/jbm.a.35905 27643636

[B5] Aria M. Cuccurullo C. (2017). Bibliometrix : An R-tool for comprehensive science mapping analysis. J. Inf. 11 (4), 959–975. 10.1016/j.joi.2017.08.007

[B6] Arrowsmith J. Miller P. (2013). Trial watch: Phase II and phase III attrition rates 2011-2012. Nat. Rev. Drug Discov. 12 (8), 569. 10.1038/nrd4090 23903212

[B7] Baniasadi H. Polez R. T. Kimiaei E. Madani Z. Rojas O. J. Osterberg M. (2021). 3D printing and properties of cellulose nanofibrils-reinforced quince seed mucilage bio-inks. Int. J. Biol. Macromol. 192, 1098–1107. 10.1016/j.ijbiomac.2021.10.078 34666132

[B8] Bertassoni L. E. Cardoso J. C. Manoharan V. Cristino A. L. Bhise N. S. Araujo W. A. (2014). Direct-write bioprinting of cell-laden methacrylated gelatin hydrogels. Biofabrication 6 (2), 024105. 10.1088/1758-5082/6/2/024105 24695367 PMC4040163

[B9] Berthiaume F. Moghe P. V. TonerM. Yarmush M. L. (1996). Effect of extracellular matrix topology on cell structure, function, and physiological responsiveness: Hepatocytes cultured in a sandwich configuration. FASEB J. 10 (13), 1471–1484. 10.1096/fasebj.10.13.8940293 8940293

[B10] Bhatia S. N. Underhill G. H. Zaret K. S. Fox I. J. (2014). Cell and tissue engineering for liver disease. Sci. Transl. Med. 6 (245), 245sr2. 10.1126/scitranslmed.3005975 25031271 PMC4374645

[B11] Bhise N. S. Manoharan V. Massa S. Tamayol A. Ghaderi M. Miscuglio M. (2016). A liver-on-a-chip platform with bioprinted hepatic spheroids. Biofabrication 8 (1), 014101. 10.1088/1758-5090/8/1/014101 26756674

[B12] Billiet T. Gevaert E. De Schryver T. Cornelissen M. Dubruel P. (2014). The 3D printing of gelatin methacrylamide cell-laden tissue-engineered constructs with high cell viability. Biomaterials 35 (1), 49–62. 10.1016/j.biomaterials.2013.09.078 24112804

[B13] Cuvellier M. Ezan F. Oliveira H. Rose S. Fricain J. C. Langouet S. (2021). 3D culture of HepaRG cells in GelMa and its application to bioprinting of a multicellular hepatic model. Biomaterials 269, 120611. 10.1016/j.biomaterials.2020.120611 33385685

[B14] Drost J. Clevers H. (2018). Organoids in cancer research. Nat. Rev. Cancer 18 (7), 407–418. 10.1038/s41568-018-0007-6 29692415

[B15] Gori M. Giannitelli S. M. Torre M. Mozetic P. Abbruzzese F. Trombetta M. (2020). Biofabrication of hepatic constructs by 3D bioprinting of a cell-laden thermogel: An effective tool to assess drug-induced hepatotoxic response. Adv. Healthc. Mat. 9 (21), e2001163. 10.1002/adhm.202001163 32940019

[B16] Grix T. Ruppelt A. Thomas A. Amler A. K. Noichl B. P. Lauster R. (2018). Bioprinting perfusion-enabled liver equivalents for advanced organ-on-a-chip applications. Genes (Basel) 9 (4), E176. 10.3390/genes9040176 PMC5924518 29565814

[B17] Gu Z. Fu J. Lin H. He Y. (2020). Development of 3D bioprinting: From printing methods to biomedical applications. Asian J. Pharm. Sci. 15 (5), 529–557. 10.1016/j.ajps.2019.11.003 33193859 PMC7610207

[B18] Guguen-Guillouzo C. Corlu A. Guillouzo A. (2010). Stem cell-derived hepatocytes and their use in toxicology. Toxicology 270 (1), 3–9. 10.1016/j.tox.2009.09.019 19815049

[B19] Heinrich M. A. Liu W. Jimenez A. Yang J. Akpek A. Liu X. (2019). 3D bioprinting: From benches to translational applications. Small 15 (23), e1805510. 10.1002/smll.201805510 31033203 PMC6752725

[B20] Hong S. Song J. M. (2021). A 3D cell printing-fabricated HepG2 liver spheroid model for high-content *in situ* quantification of drug-induced liver toxicity. Biomater. Sci. 9 (17), 5939–5950. 10.1039/d1bm00749a 34318795

[B21] Jeon H. Kang K. Park S. A. Kim W. D. Paik S. S. Lee S. H. (2017). Generation of multilayered 3D structures of HepG2 cells using a bio-printing technique. Gut Liver 11 (1), 121–128. 10.5009/gnl16010 27559001 PMC5221869

[B22] Jeong W. Kim M. K. Kang H. W. (2021). Effect of detergent type on the performance of liver decellularized extracellular matrix-based bio-inks. J. Tissue Eng. 12, 2041731421997091. 10.1177/2041731421997091 33717429 PMC7919203

[B23] Jia W. Gungor-Ozkerim P. S. Zhang Y. S. Yue K. Zhu K. Liu W. (2016). Direct 3D bioprinting of perfusable vascular constructs using a blend bioink. Biomaterials 106, 58–68. 10.1016/j.biomaterials.2016.07.038 27552316 PMC5300870

[B24] Kang D. Hong G. An S. Jang I. Yun W. S. Shim J. H. (2020). Bioprinting of multiscaled hepatic lobules within a highly vascularized construct. Small 16 (13), e1905505. 10.1002/smll.201905505 32078240

[B25] Kang H. K. Sarsenova M. Kim D. H. Kim M. S. Lee J. Y. Sung E. A. (2021). Establishing a 3D *in vitro* hepatic model mimicking physiologically relevant to *in vivo* state. Cells 10 (5), 1268. 10.3390/cells10051268 34065411 PMC8161177

[B26] Kang K. Kim Y. Jeon H. Lee S. B. Kim J. S. Park S. A. (2018). Three-dimensional bioprinting of hepatic structures with directly converted hepatocyte-like cells. Tissue Eng. Part A 24 (7-8), 576–583. 10.1089/ten.TEA.2017.0161 28726547

[B27] Kim M. K. Jeong W. Lee S. M. Kim J. B. Jin S. Kang H. W. (2020). Decellularized extracellular matrix-based bio-ink with enhanced 3D printability and mechanical properties. Biofabrication 12 (2), 025003. 10.1088/1758-5090/ab5d80 31783385

[B28] Lee H. Chae S. Kim J. Y. Han W. Kim J. Choi Y. (2019). Cell-printed 3D liver-on-a-chip possessing a liver microenvironment and biliary system. Biofabrication 11 (2), 025001. 10.1088/1758-5090/aaf9fa 30566930

[B29] Lee H. Han W. Kim H. Ha D. H. Jang J. Kim B. S. (2017). Development of liver decellularized extracellular matrix bioink for three-dimensional cell printing-based liver tissue engineering. Biomacromolecules 18 (4), 1229–1237. 10.1021/acs.biomac.6b01908 28277649

[B30] Lee H. Kim J. Choi Y. Cho D. W. (2020). Application of gelatin bioinks and cell-printing technology to enhance cell delivery capability for 3D liver fibrosis-on-a-chip development. ACS Biomater. Sci. Eng. 6 (4), 2469–2477. 10.1021/acsbiomaterials.9b01735 33455331

[B31] Lewis P. L. Green R. M. Shah R. N. (2018). 3D-printed gelatin scaffolds of differing pore geometry modulate hepatocyte function and gene expression. Acta Biomater. 69, 63–70. 10.1016/j.actbio.2017.12.042 29317370 PMC5831494

[B32] Lewis P. L. Yan M. Su J. Shah R. N. (2019). Directing the growth and alignment of biliary epithelium within extracellular matrix hydrogels. Acta Biomater. 85, 84–93. 10.1016/j.actbio.2018.12.039 30590182 PMC6768828

[B33] Lorsch J. R. Collins F. S. Lippincott-Schwartz J. (2014). Cell Biology. Fixing problems with cell lines. Science 346 (6216), 1452–1453. 10.1126/science.1259110 25525228 PMC5101941

[B34] Ma L. Wu Y. Li Y. Aazmi A. Zhou H. Zhang B. (2020). Current advances on 3D-bioprinted liver tissue models. Adv. Healthc. Mat. 9 (24), e2001517. 10.1002/adhm.202001517 33073522

[B35] Ma X. Yu C. Wang P. Xu W. Wan X. Lai C. S. E. (2018). Rapid 3D bioprinting of decellularized extracellular matrix with regionally varied mechanical properties and biomimetic microarchitecture. Biomaterials 185, 310–321. 10.1016/j.biomaterials.2018.09.026 30265900 PMC6186504

[B36] Mao Q. Wang Y. Li Y. Juengpanich S. Li W. Chen M. (2020). Fabrication of liver microtissue with liver decellularized extracellular matrix (dECM) bioink by digital light processing (DLP) bioprinting. Mat. Sci. Eng. C Mat. Biol. Appl. 109, 110625. 10.1016/j.msec.2020.110625 32228893

[B37] Mazzocchi A. Devarasetty M. Huntwork R. Soker S. Skardal A. (2018). Optimization of collagen type I-hyaluronan hybrid bioink for 3D bioprinted liver microenvironments. Biofabrication 11 (1), 015003. 10.1088/1758-5090/aae543 30270846 PMC8008502

[B38] Nguyen D. G. Funk J. Robbins J. B. Crogan-Grundy C. Presnell S. C. Singer T. (2016). Bioprinted 3D primary liver tissues allow assessment of organ-level response to clinical drug induced toxicity *in vitro* . PLoS One 11 (7), e0158674. 10.1371/journal.pone.0158674 27387377 PMC4936711

[B39] Norona L. M. Nguyen D. G. Gerber D. A. Presnell S. C. Mosedale M. Watkins P. B. (2019). Bioprinted liver provides early insight into the role of Kupffer cells in TGF-β1 and methotrexate-induced fibrogenesis. PLoS One 14 (1), e0208958. 10.1371/journal.pone.0208958 30601836 PMC6314567

[B40] Ozbolat I. T. Hospodiuk M. (2016). Current advances and future perspectives in extrusion-based bioprinting. Biomaterials 76, 321–343. 10.1016/j.biomaterials.2015.10.076 26561931

[B41] Pan C. Kumar C. Bohl S. Klingmueller U. Mann M. (2009). Comparative proteomic phenotyping of cell lines and primary cells to assess preservation of cell type-specific functions. Mol. Cell. Proteomics 8 (3), 443–450. 10.1074/mcp.M800258-MCP200 18952599 PMC2649808

[B42] Parsa S. Gupta M. Loizeau F. Cheung K. C. (2010). Effects of surfactant and gentle agitation on inkjet dispensing of living cells. Biofabrication 2 (2), 025003. 10.1088/1758-5082/2/2/025003 20811131

[B43] Perez R. A. Kim H. W. (2015). Core-shell designed scaffolds for drug delivery and tissue engineering. Acta Biomater. 21, 2–19. 10.1016/j.actbio.2015.03.013 25792279

[B44] Rastogi P. Kandasubramanian B. (2019). Review of alginate-based hydrogel bioprinting for application in tissue engineering. Biofabrication 11 (4), 042001. 10.1088/1758-5090/ab331e 31315105

[B45] Rouwkema J. Khademhosseini A. (2016). Vascularization and angiogenesis in tissue engineering: Beyond creating static networks. Trends Biotechnol. 34 (9), 733–745. 10.1016/j.tibtech.2016.03.002 27032730

[B46] Sun L. Yang H. Wang Y. Zhang X. Jin B. Xie F. (2020). Application of a 3D bioprinted hepatocellular carcinoma cell model in antitumor drug research. Front. Oncol. 10, 878. 10.3389/fonc.2020.00878 32582546 PMC7283506

[B47] Sun W. Starly B. Daly A. C. Burdick J. A. Groll J. Skeldon G. (2020). The bioprinting roadmap. Biofabrication 12 (2), 022002. 10.1088/1758-5090/ab5158 32031083

[B48] Swift B. Pfeifer N. D. Brouwer K. L. (2010). Sandwich-cultured hepatocytes: An *in vitro* model to evaluate hepatobiliary transporter-based drug interactions and hepatotoxicity. Drug Metab. Rev. 42 (3), 446–471. 10.3109/03602530903491881 20109035 PMC3097390

[B49] Taymour R. Kilian D. Ahlfeld T. Gelinsky M. Lode A. (2021). 3D bioprinting of hepatocytes: Core-shell structured co-cultures with fibroblasts for enhanced functionality. Sci. Rep. 11 (1), 5130. 10.1038/s41598-021-84384-6 33664366 PMC7933206

[B50] Treyer A. Musch A. (2013). Hepatocyte polarity. Compr. Physiol. 3 (1), 243–287. 10.1002/cphy.c120009 23720287 PMC3697931

[B51] van Grunsven L. A. (2017). 3D *in vitro* models of liver fibrosis. Adv. Drug Deliv. Rev. 121, 133–146. 10.1016/j.addr.2017.07.004 28697953

[B52] Wu Y. Wenger A. Golzar H. Tang X. S. (2020). 3D bioprinting of bicellular liver lobule-mimetic structures via microextrusion of cellulose nanocrystal-incorporated shear-thinning bioink. Sci. Rep. 10 (1), 20648. 10.1038/s41598-020-77146-3 33244046 PMC7691334

[B53] Xie F. Sun L. Pang Y. Xu G. Jin B. Xu H. (2021). Three-dimensional bio-printing of primary human hepatocellular carcinoma for personalized medicine. Biomaterials 265, 120416. 10.1016/j.biomaterials.2020.120416 33007612

[B54] Xie F. Xiao Y. Chen M. (2020). Three-dimensional bioprinted liver tissue for transplantation: Hope or hype? Hepatobiliary Surg. Nutr. 9 (6), 788–790. 10.21037/hbsn-20-549 33299836 PMC7720053

[B55] Yan Y. Wang X. Pan Y. Liu H. Cheng J. Xiong Z. (2005). Fabrication of viable tissue-engineered constructs with 3D cell-assembly technique. Biomaterials 26 (29), 5864–5871. 10.1016/j.biomaterials.2005.02.027 15949552

[B56] Yang H. Sun L. Pang Y. Hu D. Xu H. Mao S. (2021). Three-dimensional bioprinted hepatorganoids prolong survival of mice with liver failure. Gut 70 (3), 567–574. 10.1136/gutjnl-2019-319960 32434830 PMC7873413

[B57] Yu C. Ma X. Zhu W. Wang P. Miller K. L. Stupin J. (2019). Scanningless and continuous 3D bioprinting of human tissues with decellularized extracellular matrix. Biomaterials 194, 1–13. 10.1016/j.biomaterials.2018.12.009 30562651 PMC6339581

[B58] Zhong C. Xie H-Y. Zhou L. Xu X. Zheng S-S. (2016). Human hepatocytes loaded in 3D bioprinting generate mini-liver. Hepatobiliary Pancreat. Dis. Int. 15 (5), 512–518. 10.1016/s1499-3872(16)60119-4 27733321

