# Supplementary material for: Current trends and research topics regarding liver 3D bioprinting: A bibliometric analysis research
Source: Front Cell Dev Biol. 2022 Nov 28;10:1047524. doi: 10.3389/fcell.2022.1047524 (PMC9742412; doi:10.3389/fcell.2022.1047524)
Supplement: Supplementary file 1 [file Table1.DOCX]

**Table S1: A summary of the key words and search strategy to identify the articles from the Clarivate Analytics Web of Science Core Collection database**

**#1:** ALL=((3D print) OR (3D printing) OR (three-dimensional printing) OR (3D Bioprinting) OR (3D-printing) OR (3D model) OR (3D technique) OR (3D structures) OR (3D printer) OR (3D printed) OR (3D hydrogel scaffolds) OR (3D scaffold) OR (3DP) OR (3D bioprinted))

**#2:** ALL=((liver) OR (hepatology) OR (hepatocyte) OR (hepatic cell) OR (liver cell) OR (HepG2) OR (HepaRG) or (hiHep) OR (hepatocellular) OR (Liver Neoplasm) OR (Hepatic Neoplasm) OR (Liver Cancer) OR (Hepatic Cancer) OR (Liver tumor) OR (Hepatic tumor) OR (Hepatocellular Cancer) OR (Hepatocellular Carcinoma) OR (HCC) OR (Hepatoma) OR (Liver malignancy) OR (liver malignant tumor) OR (hepatotoxicity) OR (gastroenterology) OR (HOC))

**#3:** #1 AND #2

Timespan= From January 1991 to January 2022.
