# Supplementary material for: Current trends and research topics regarding liver 3D bioprinting: A bibliometric analysis research
Source: Front Cell Dev Biol. 2022 Nov 28;10:1047524. doi: 10.3389/fcell.2022.1047524 (PMC9742412; doi:10.3389/fcell.2022.1047524)
Supplement: Supplementary file 2 [file Table2.DOCX]

| Rank | Article | TC |
| --- | --- | --- |
| 1 | Billiet T, et al. The 3D printing of gelatin methacrylamide cell-laden tissue-engineered constructs with high cell viability. Biomaterials. 2014;35(1):49-62. | 565 |
| 2 | Ma X, et al. Deterministically patterned biomimetic human iPSC-derived hepatic model via rapid 3D bioprinting. Proc Natl Acad Sci U S A. 2016;113(8):2206-11. | 389 |
| 3 | Bertassoni LE, et al. Direct-write bioprinting of cell-laden methacrylated gelatin hydrogels. Biofabrication. 2014;6(2):024105. | 333 |
| 4 | Bhise NS, et al. A liver-on-a-chip platform with bioprinted hepatic spheroids. Biofabrication. 2016;8(1):014101. | 275 |
| 5 | Skardal A, et al. Bioprinting vessel-like constructs using hyaluronan hydrogels crosslinked with tetrahedral polyethylene glycol tetracrylates. Biomaterials. 2010;31(24):6173-81. | 273 |
| 6 | Skardal A, et al. Photocrosslinkable Hyaluronan-Gelatin Hydrogels for Two-Step Bioprinting. Tissue Eng Part A. 2010;16(8):2675-85. | 270 |
| 7 | Wang X, et al. Generation of three-dimensional hepatocyte/gelatin structures with rapid prototyping system. Tissue Eng. 2006;12(1):83-90. | 247 |
| 8 | Faulkner-Jones A, et al. Bioprinting of human pluripotent stem cells and their directed differentiation into hepatocyte-like cells for the generation of mini-livers in 3D. Biofabrication. 2015;7(4):044102. | 227 |
| 9 | Skardal A, et al. A hydrogel bioink toolkit for mimicking native tissue biochemical and mechanical properties in bioprinted tissue constructs. Acta Biomater. 2015;25:24-34. | 212 |
| 10 | Yan Y, et al. Fabrication of viable tissue-engineered constructs with 3D cell-assembly technique. Biomaterials. 2005;26(29):5864-71. | 191 |
| 11 | Nguyen DG, et al. Bioprinted 3D Primary Liver Tissues Allow Assessment of Organ-Level Response to Clinical Drug Induced Toxicity In Vitro. PLoS One. 2016;11(7):e0158674. | 188 |
| 12 | Lee H, et al. One-step fabrication of an organ-on-a-chip with spatial heterogeneity using a 3D bioprinting technology. Lab Chip. 2016;16(14):2618-25. | 152 |
| 13 | Chang R, et al. Biofabrication of a three-dimensional liver micro-organ as an in vitro drug metabolism model. Biofabrication. 2010;2(4):045004. | 152 |
| 14 | Lee H, et al. Development of Liver Decellularized Extracellular Matrix Bioink for Three-Dimensional Cell Printing-Based Liver Tissue Engineering. Biomacromolecules. 2017 Apr;18(4):1229-1237. | 132 |
| 15 | Ying GL, et al. Aqueous Two-Phase Emulsion Bioink-Enabled 3D Bioprinting of Porous Hydrogels. Adv Mater. 2018;30(50):e1805460. | 120 |
| 16 | Lee JW, et al. Development of a 3D cell printed construct considering angiogenesis for liver tissue engineering. Biofabrication. 2016;8(1):015007. | 117 |
| 17 | Snyder JE, et al. Bioprinting cell-laden matrigel for radioprotection study of liver by pro-drug conversion in a dual-tissue microfluidic chip. Biofabrication. 2011;3(3):034112. | 117 |
| 18 | Matsusaki M, et al. Three-Dimensional Human Tissue Chips Fabricated by Rapid and Automatic Inkjet Cell Printing. Adv Healthc Mater. 2013;2(4):534-9. | 107 |
| 19 | Yu C, et al. Scanningless and continuous 3D bioprinting of human tissues with decellularized extracellular matrix. Biomaterials. 2019;194:1-13. | 89 |
| 20 | Ma X, et al. Rapid 3D bioprinting of decellularized extracellular matrix with regionally varied mechanical properties and biomimetic microarchitecture. Biomaterials. 2018;185:310-321. | 81 |
| 21 | Lee HJ, et al. A New Approach for Fabricating Collagen/ECM-Based Bioinks Using Preosteoblasts and Human Adipose Stem Cells. Adv Healthc Mater. 2015;4(9):1359-68. | 80 |
| 22 | Mazzocchi A, et al. Optimization of collagen type I-hyaluronan hybrid bioink for 3D bioprinted liver microenvironments. Biofabrication. 2018;11(1):015003. | 78 |
| 23 | Norona LM, et al. Modeling Compound-Induced Fibrogenesis In Vitro Using Three-Dimensional Bioprinted Human Liver Tissues. Toxicol Sci. 2016;154(2):354-367. | 77 |
| 24 | Massa S, et al. Bioprinted 3D vascularized tissue model for drug toxicity analysis. Biomicrofluidics. 2017;11(4):044109. | 68 |
| 25 | Grix T, et al. Bioprinting Perfusion-Enabled Liver Equivalents for Advanced Organ-on-a-Chip Applications. Genes (Basel). 2018;9(4):176. | 60 |
| 26 | Hiller T, et al. Generation of a 3D Liver Model Comprising Human Extracellular Matrix in an Alginate/Gelatin-Based Bioink by Extrusion Bioprinting for Infection and Transduction Studies. Int J Mol Sci. 2018;19(10):3129. | 53 |
| 27 | Parsa S, et al. Effects of surfactant and gentle agitation on inkjet dispensing of living cells. Biofabrication. 2010;2(2):025003. | 51 |
| 28 | Lee H, et al. Cell-printed 3D liver-on-a-chip possessing a liver microenvironment and biliary system. Biofabrication. 2019;11(2):025001. | 45 |
| 29 | Jeon H, et al. Generation of Multilayered 3D Structures of HepG2 Cells Using a Bio-printing Technique. Gut Liver. 2017;11(1):121-128. | 45 |
| 30 | Skardal A, et al. Bioprinting Cellularized Constructs Using a Tissue-specific Hydrogel Bioink. J Vis Exp. 2016;(110):e53606. | 39 |
| 31 | Mao Q, et al. Fabrication of liver microtissue with liver decellularized extracellular matrix (dECM) bioink by digital light processing (DLP) bioprinting. Mater Sci Eng C Mater Biol Appl. 2020;109:110625. | 37 |
| 32 | Goulart E, et al. 3D bioprinting of liver spheroids derived from human induced pluripotent stem cells sustain liver function and viability in vitro. Biofabrication. 2019;12(1):015010. | 34 |
| 33 | Jian H, et al. Dipeptide Self-Assembled Hydrogels with Tunable Mechanical Properties and Degradability for 3D Bioprinting. ACS Appl Mater Interfaces. 2019;11(50):46419-46426. | 31 |
| 34 | Norona LM, et al. Bioprinted liver provides early insight into the role of Kupffer cells in TGF-beta 1 and methotrexate-induced fibrogenesis. PLoS One. 2019;14(1):e0208958. | 31 |
| 35 | Wang L, et al. Iterative feedback bio-printing-derived cell-laden hydrogel scaffolds with optimal geometrical fidelity and cellular controllability. Sci Rep. 2018;8(1):2802. | 31 |
| 36 | Kim Y, et al. Three-dimensional (3D) printing of mouse primary hepatocytes to generate 3D hepatic structure. Ann Surg Treat Res. 2017;92(2):67-72. | 30 |
| 37 | Kang K, et al. Three-Dimensional Bioprinting of Hepatic Structures with Directly Converted Hepatocyte-Like Cells. Tissue Eng Part A. 2018;24(7-8):576-583. | 26 |
| 38 | Kim MK, et al. Decellularized extracellular matrix-based bio-ink with enhanced 3D printability and mechanical properties. Biofabrication. 2020;12(2):025003. | 24 |
| 39 | Kang D, et al. Bioprinting of Multiscaled Hepatic Lobules within a Highly Vascularized Construct. Small. 2020;16(13):e1905505. | 24 |
| 40 | Kang D, et al. Pre-set extrusion bioprinting for multiscale heterogeneous tissue structure fabrication. Biofabrication. 2018;10(3):035008. | 24 |
| 41 | Zhong C, et al. Human hepatocytes loaded in 3D bioprinting generate mini-liver. Hepatobiliary Pancreat Dis Int. 2016;15(5):512-518. | 24 |
| 42 | Yang H, et al. Three-dimensional bioprinted hepatorganoids prolong survival of mice with liver failure. Gut. 2021;70(3):567-574. | 23 |
| 43 | Arai K, et al. Fabrication of 3D-culture platform with sandwich architecture for preserving liver-specific functions of hepatocytes using 3D bioprinter. J Biomed Mater Res A. 2017;105(6):1583-1592. | 21 |
| 44 | Ahn J, et al. Human three-dimensional in vitro model of hepatic zonation to predict zonal hepatotoxicity. J Biol Eng. 2019;13:22. | 18 |
| 45 | Damiati S, et al. Embedded Disposable Functionalized Electrochemical Biosensor with a 3D-Printed Flow Cell for Detection of Hepatic Oval Cells (HOCs). Genes (Basel). 2018;9(2):89. | 17 |
| 46 | Sun L, et al. Application of a 3D Bioprinted Hepatocellular Carcinoma Cell Model in Antitumor Drug Research. Front Oncol. 2020;10:878. | 16 |
| 47 | Li Y, et al. 3D bioprinting of hepatoma cells and application with microfluidics for pharmacodynamic test of Metuzumab. Biofabrication. 2019;11(3):034102. | 16 |
| 48 | Rimington RP, et al. Feasibility and Biocompatibility of 3D-Printed Photopolymerized and Laser Sintered Polymers for Neuronal, Myogenic, and Hepatic Cell Types. Macromol Biosci. 2018;18(7):e1800113. | 16 |
| 49 | Kim Y, et al. Prolongation of liver-specific function for primary hepatocytes maintenance in 3D printed architectures. Organogenesis. 2018;14(1):1-12. | 16 |
| 50 | Gori M, et al. Biofabrication of Hepatic Constructs by 3D Bioprinting of a Cell-Laden Thermogel: An Effective Tool to Assess Drug-Induced Hepatotoxic Response. Adv Healthc Mater. 2020:e2001163. | 14 |
| 51 | Xie F, et al. Three-dimensional bio-printing of primary human hepatocellular carcinoma for personalized medicine. Biomaterials. 2021;265:120416. | 13 |
| 52 | Hwang HH, et al. High throughput direct 3D bioprinting in multiwell plates. Biofabrication. 2020; 30, 2005324. | 12 |
| 53 | Yanagi Y, et al. In vivo and ex vivo methods of growing a liver bud through tissue connection. Sci Rep. 2017;7(1):14085. | 12 |
| 54 | Mao S, et al. Bioprinting of patient-derivedin vitrointrahepatic cholangiocarcinoma tumor model: establishment, evaluation and anti-cancer drug testing. Biofabrication. 2020;12(4):045014. | 11 |
| 55 | Jeon S, et al. High-Precision 3D Bio-Dot Printing to Improve Paracrine Interaction between Multiple Types of Cell Spheroids. Adv Funct Mater. 2020;30,2005324. | 11 |
| 56 | Taymour R, et al. 3D bioprinting of hepatocytes: core-shell structured co-cultures with fibroblasts for enhanced functionality. Sci Rep. 2021;11(1):5130. | 10 |
| 57 | Cuvellier M, et al. 3D culture of HepaRG cells in GelMa and its application to bioprinting of a multicellular hepatic model. Biomaterials. 2021;269:120611. | 9 |
| 58 | Jeffries GDM, et al. 3D micro-organisation printing of mammalian cells to generate biological tissues. Sci Rep. 2020;10(1):19529. | 8 |
| 59 | He H, et al. Temperature-programmable and enzymatically solidifiable gelatin-based bioinks enable facile extrusion bioprinting. Biofabrication. 2020;12(4):045003. | 8 |
| 60 | Wu Y, et al. 3D bioprinting of bicellular liver lobule-mimetic structures via microextrusion of cellulose nanocrystal-incorporated shear-thinning bioink. Sci Rep. 2020;10(1):20648. | 7 |
| 61 | Lee H, et al. Application of Gelatin Bioinks and Cell-Printing Technology to Enhance Cell Delivery Capability for 3D Liver Fibrosis-on-a-Chip Development. ACS Biomater Sci Eng. 2020;6(4):2469-2477. | 7 |
| 62 | Leva V, et al. Direct Laser Printing of Liver Cells on Porous Collagen Scaffolds. Journal of Laser Micro/Nanoengineering. 2018;13(3):234-237 | 5 |
| 63 | Yu S, et al. Deconvolution of Images from 3D Printed Cells in Layers on a Chip. Biotechnol Prog. 2018;34(2):445-454. | 4 |
| 64 | Lee JS, et al. Development of hepatic blocks using human adipose tissue-derived stem cells through three-dimensional cell printing techniques. J Mater Chem B. 2017;5(5):1098-1107. | 4 |
| 65 | Hong G, et al. Production of Multiple Cell-Laden Microtissue Spheroids with a Biomimetic Hepatic-Lobule-Like Structure. Adv Mater. 2021;33(36):e2102624. | 3 |
| 66 | Ide I, et al. A novel evaluation method for determining drug-induced hepatotoxicity using 3D bio-printed human liver tissue. Toxicol Mech Methods. 2020;30(3):189-196. | 3 |
| 67 | Baniasadi, H, et al. 3D printing and properties of cellulose nanofibrils-reinforced quince seed mucilage bio-inks. Int J Biol Macromol. 2021;192:1098-1107. | 1 |
| 68 | Hong S, et al. A 3D cell printing-fabricated HepG2 liver spheroid model for high-content in situ quantification of drug-induced liver toxicity. Biomater Sci. 2021;9(17):5939-5950. | 1 |
| 69 | Kang HK, et al. Establishing a 3D In Vitro Hepatic Model Mimicking Physiologically Relevant to In Vivo State. Cells. 2021;10(5):1268. | 1 |
| 70 | Jeong W, et al.Effect of detergent type on the performance of liver decellularized extracellular matrix-based bio-inks. J Tissue Eng. 2021;12:2041731421997091. | 1 |
| 71 | Hamada T, et al. Bile duct reconstruction using scaffold-free tubular constructs created by Bio-3D printer. Regen Ther. 2021;16:81-89. | 0 |
