# Supplementary material for: Current trends and research topics regarding liver 3D bioprinting: A bibliometric analysis research
Source: Front Cell Dev Biol. 2022 Nov 28;10:1047524. doi: 10.3389/fcell.2022.1047524 (PMC9742412; doi:10.3389/fcell.2022.1047524)
Supplement: Supplementary file 3 [file Table3.DOCX]

**Table S3: Countries or regions that published articles.**

| Country | Publication | TC | TC/Publication |
| --- | --- | --- | --- |
| USA | 27 | 3039 | 112.56 |
| SOUTH KOREA | 22 | 833 | 37.86 |
| CHINA | 16 | 916 | 57.25 |
| JAPAN | 5 | 143 | 28.60 |
| GERMANY | 4 | 140 | 35.00 |
| UK | 4 | 593 | 148.25 |
| CANADA | 3 | 59 | 19.67 |
| SAUDI ARABIA | 3 | 360 | 120.00 |
| BRAZIL | 2 | 367 | 183.50 |
| CHILE | 2 | 343 | 171.50 |
| ITALY | 2 | 82 | 41.00 |
| AUSTRALIA | 1 | 333 | 333.00 |
| AUSTRIA | 1 | 17 | 17.00 |
| BELGIUM | 1 | 565 | 565.00 |
| CZECH REPUBLIC | 1 | 14 | 14.00 |
| FINLAND | 1 | 1 | 1.00 |
| FRANCE | 1 | 9 | 9.00 |
| GREECE | 1 | 5 | 5.00 |
| INDIA | 1 | 333 | 333.00 |
| IRAQ | 1 | 17 | 17.00 |
| KAZAKHSTAN | 1 | 1 | 1.00 |
| NEPAL | 1 | 120 | 120.00 |
| NETHERLANDS | 1 | 14 | 14.00 |
| SWEDEN | 1 | 8 | 8.00 |
| SWITZERLAND | 1 | 188 | 188.00 |

TC: total citation
